# Digital Dementia Education and Training for Informal Carers: A Scoping Review

**DOI:** 10.1111/jocn.17817

**Published:** 2025-05-28

**Authors:** Di Huang, Dongjun Wu, Wendy Moyle

**Affiliations:** ^1^ School of Nursing and Midwifery Griffith University Brisbane Queensland Australia

**Keywords:** dementia care technology, dementia education, digital tools, informal carers, nursing education, psychological support, scoping review

## Abstract

**Introduction:**

Dementia education is critical for equipping informal carers with the necessary skills and knowledge to provide effective care. However, delivering comprehensive and accessible education remains challenging, particularly in resource‐limited settings. Digital tools have emerged as innovative solutions to bridge these gaps, yet limited attention has been given to how such interventions are implemented in diverse caregiving contexts.

**Aim:**

This scoping review aims to identify, synthesise and critically map the implementation strategies employed in digital education programmes designed for informal carers of people living with dementia. It also explores the contextual challenges and enabling factors associated with adopting and delivering such interventions across different care settings.

**Method:**

A scoping review was conducted following the PRISMA‐ScR and JBI guidelines. The study adheres to the PRISMA‐ScR checklist to ensure transparent and comprehensive reporting. Comprehensive searches were performed in eleven databases, covering studies published up to February 2024. Out of 2747 identified records, duplicates were removed and studies were screened based on predefined inclusion criteria. Ultimately, 18 studies were included in the review.

**Results:**

The findings revealed three core areas: (a) types of digital tools used, including online platforms, video conferencing, mobile applications and SMS services; (b) educational domains addressed, such as knowledge and skills, psychological support, communication and social interaction, and environmental and technological adaptation; and (c) implementation strategies and outcomes, highlighting personalisation, cultural sensitivity and technological adaptability, which improved knowledge, emotional well‐being and carer self‐efficacy. Based on these findings, a theoretical framework was developed that proposed a progressive model—Foundational, Competency and Growth—that aligns digital tools with carers' evolving needs and caregiving stages.

**Conclusion:**

The scoping review demonstrates the potential of digital tools in informal carers' dementia education. To optimise their effectiveness, educational strategies should integrate active, adaptive and culturally tailored approaches, addressing carers' needs at both individual and systemic levels. Future research should explore the scalability, inclusivity and long‐term impact of digital dementia education programmes to enhance carer support and patient outcomes.

**Summary**

- This scoping review identifies key implementation strategies in digital dementia education for informal carers, emphasising personalisation, cultural relevance, and technological adaptability.
- It outlines four core educational domains: knowledge and skills, psychological support, communication and interaction, and environmental and technological adaptation.
- A conceptual framework is proposed that classifies digital tools into foundational, competency, and growth levels, aligning with carers' evolving needs throughout the care continuum.

## 1 Introduction

Dementia is a progressive and debilitating syndrome characterised by a decline in cognitive functions such as memory, reasoning and social abilities, surpassing the typical effects of aging (Hermann and Zerr 2022; Reuben et al. 2024). Globally, over 55 million individuals currently live with dementia, with nearly 10 million new cases diagnosed annually (Li et al. 2024). By 2025, this number is projected to rise to 150 million (Wimo et al. 2023). This highlights dementia as a significant global public health challenge, placing immense strain not only on individuals but also on their families and healthcare systems (Aranda et al. 2021).

Approximately 83% of dementia caregiving responsibilities are shouldered by informal carers, that is, family members, friends or neighbours who provide essential daily care without formal training or financial compensation within a home environment (*Alzheimer's and Dementia* 2023; Zimami and Darwish 2024). Informal carers, also called unpaid carers, play a critical yet often underappreciated role in supporting individuals with dementia (Fernandez Cajavilca and Sadarangani 2024; Happ et al. 2024). Their responsibilities encompass a wide range of tasks, including grocery shopping, medication management, overseeing financial and legal matters, preventing wandering, and assisting with both basic and complex daily activities. The responsibility of caring for a person with dementia entails profound emotional, financial and physical burdens, which often disrupt family dynamics and well‐being (Liu et al. 2022).

The burden is particularly acute in low‐ and middle‐income countries, where formal dementia care services are scarce, and informal carers often lack adequate knowledge of dementia symptoms, caregiving strategies and effective management techniques (Ainamani et al. 2020; Fam et al. 2019; Ho et al. 2024). Without adequate education and support, these carers face significant challenges, which may lead to increased stress, poor caregiving outcomes, and diminished quality of life for patients and carers (Turró‐Garriga et al. 2021). These gaps underscore the urgent need for accessible, culturally sensitive and effective dementia education programmes tailored to informal carers.

### 1.1 Digital Education and Training

Advances in information and communication technologies (ICT) have positioned digital education as a transformative solution to address the educational needs of informal carers in dementia care (Fan et al. 2023). For example, the iSupport program has demonstrated measurable reductions in carer stress and improved caregiving skills through its modular structure and interactive features (Masterson‐Algar et al. 2022). Digital education and Training (Kleib et al. 2023), including online courses, mobile applications, telehealth services, and virtual reality tools, provide scalable, flexible and accessible learning opportunities. These tools offer tailored content designed to enhance carers' knowledge, caregiving skills and psychological resilience, enabling them to manage the complexities of dementia care better (Wichmann et al. 2021).

Digital education shows several advantages over traditional face‐to‐face training models, particularly in overcoming geographic, temporal and resource‐related barriers. For example, programmes such as iSupport (Sani et al. 2024) and CARES (Collins‐Pisano et al. 2024) exemplify this potential by integrating evidence‐based content, interactive modules and real‐time feedback, providing practical strategies for carers to manage behavioural symptoms, improve communication and address emotional challenges. In addition, the self‐paced nature of digital platforms makes the information accessible for carers. These tools have demonstrated positive outcomes, including enhanced self‐efficacy, reduced carer stress and improved care quality (Scerbe et al. 2023).

However, the implementation of digital education for informal carers faces significant challenges. Digital literacy remains a major barrier, particularly for older carers or those in resource‐limited settings. For instance, studies have shown that older informal carers often struggle to navigate complex mobile application interfaces, resulting in low engagement rates (Petrovic and Gaggioli 2020). To address this, interventions like CuidaTEXT have adopted simplified SMS‐based designs, demonstrating higher usability and engagement (Perales‐Puchalt et al. 2024). While high‐fidelity tools such as virtual reality (VR) and augmented reality (AR) provide immersive educational experiences, their complexity often limits accessibility (Plotzky et al. 2021; Zhi et al. 2024). Conversely, low‐complexity tools like video conferencing and telehealth have proven effective in low‐ and middle‐income countries by offering practical, user‐friendly solutions (Perales‐Puchalt et al. 2024; Rapley et al. 2023). However, sustained user engagement requires platforms that are intuitive, culturally sensitive and responsive to the diverse needs of informals.

Previous reviews have explored dementia training for informal carers and the general public (Han et al. 2024; Kay et al. 2023; Matsumoto et al. 2023; Zimami and Darwish 2024), and several systematic reviews have evaluated the effectiveness of digital education programmes in reducing caregiver burden and improving mental health outcomes (Egan et al. 2018; Etxeberria et al. 2021; Leng et al. 2020; Yu et al. 2023). However, limited attention has been given to the implementation strategies that underpin the successful delivery, contextual adaptation and sustainability of these interventions. To address this gap, the present scoping review maps existing evidence on implementation strategies used in digital dementia education for informal carers, identifies key challenges and enablers, and outlines implications for future intervention design and programme development.

### 1.2 Aims

Accordingly, this scoping review aims to systematically map and synthesise the current landscape of digital education and training programmes designed to support informal carers of individuals living with dementia. Specifically, it seeks to identify the digital platforms and tools employed, the caregiving needs and educational domains addressed, and the implementation strategies that facilitate their delivery and integration across diverse care settings. Consistent with the methodological purpose of a scoping review, this study does not assess intervention effects but rather identifies and summarises the outcome indicators reported in the included literature. The specific aims of this review are as follows:

1. To identify and categorise the types of digital tools and platforms used in dementia education and training programmes targeting informal carers.
2. To describe the caregiving challenges and educational domains these digital interventions address.
3. To map the implementation strategies employed in designing and delivering digital education programmes and summarise the outcome indicators reported in the included studies.

## 2 Method

### 2.1 Design

A scoping review methodology was adopted to explore digital education and training programmes for informal carers in dementia care comprehensively. This approach is particularly suited for mapping the breadth of existing research, identifying knowledge gaps and summarising available evidence on a specific topic. The review adhered to five of the six stages outlined in the framework proposed by (Arksey and O'Malley 2005), further refined by (Levac et al. 2010): (a) identifying the research question; (b) identifying relevant studies; (c) selecting studies; (d) charting the data; and (e) collating, summarising and reporting results.

In addition, we incorporated the methodological guidance of (Peters et al. 2020, 2021b), which emphasises rigorous evidence screening and selection, systematic data extraction, and effective analysis and presentation of results. This scoping review was conducted in accordance with the Preferred Reporting Items for Systematic Reviews and Meta‐Analyses Extension for Scoping Reviews (PRISMA‐ScR): Checklist and Explanation (Tricco et al. 2018) and the Joanna Briggs Institute (JBI) methodological guidance for scoping reviews (Peters et al. 2020). The PRISMA‐ScR checklist has been submitted as Table S1 to ensure transparent and comprehensive reporting.

### 2.2 Clarifying the Review Question

This scoping review systematically maps and synthesises evidence on the use and effectiveness of digital tools in dementia education and training for informal carers. The aim is to explore and identify how digital interventions address informal carers' education needs and outcomes. Three specific questions guide the review:

1. What types of digital tools and platforms are employed in dementia education and training for informal carers?
2. Which caregiving challenges and educational domains are addressed by these digital interventions?
3. What are the key implementation strategies and outcomes associated with digital dementia education programmes for informal carers?

This review defines informal carers as family members, friends or neighbours, who provide primary care and support to individuals with dementia without formal nursing training. These carers often experience significant challenges, including understanding dementia symptoms, managing patient needs and applying effective caregiving strategies. Digital education offers an innovative and scalable approach to address these challenges by leveraging online platforms, mobile applications, telehealth services and virtual reality tools. These tools are designed to provide dementia‐specific training in symptom management, caregiving skills and psychological support.

This review defines implementation strategies as the structured methods and processes used to promote the adoption, integration, and sustained delivery of digital education interventions within real‐world dementia care contexts. These strategies may involve tailoring educational content to carers' needs, enhancing accessibility through technology platforms, providing technical or psychosocial support, incorporating iterative feedback mechanisms, and ensuring cultural and contextual relevance. This operational definition is informed by implementation science frameworks (Proctor et al. 2013) and underpins the identification, extraction and synthesis of relevant data across included studies.

To address these research questions comprehensively, this scoping review focuses on studies that provide empirical evidence about using digital tools for dementia education targeting informal carers.

### 2.3 Identifying Relevant Studies

#### 2.3.1 Inclusion and Exclusion Criteria

This review includes studies published in English and Chinese (two reviewers read and write Chinese and English) since the databases' inception, encompassing all types of qualitative and quantitative research designs focused on digital dementia education and training (see Table 1).

**TABLE 1:** Inclusion and exclusion criteria for the scoping review.

Category	Inclusion criteria	Exclusion criteria
Participants	Studies targeting informal carers (family members, friends, or neighbours) who provide care without professional nursing training. Studies that included formal and informal carers were also considered, focusing on results specific to informal carers	Studies focusing on formal carers, such as healthcare professionals or those with formal caregiving training
Concept	Digital dementia education and training programmes, including online courses, mobile applications, telehealth, virtual reality and other digital platforms	Interventions not involving digital tools or those using purely non‐digital methods, such as traditional face‐to‐face training
Outcomes	Studies reporting any outcomes related to changes in carers' knowledge, attitudes, skills, psychological well‐being, or caregiving practices	Studies without specific outcomes relevant to informal carers or those focusing solely on patient outcomes
Types	Empirical studies with qualitative, quantitative, or mixed‐method designs published in peer‐reviewed journals	Non‐empirical studies, reviews, editorials, book chapters, guidelines and non‐peer‐reviewed literature
Publication date	Studies published from the databases' inception (e.g., PubMed, Scopus) up to the review's cut‐off date	Studies not published in English or Chinese

#### 2.3.2 Data Sources and Search Strategy

This review employed a comprehensive and systematic literature search to ensure the identification of relevant studies. To design a sensitive and inclusive search strategy, we utilised thematic terms encompassing both controlled vocabulary and free‐text keywords. These terms were selected based on preliminary searches and the working group's expertise. MeSH‐Browser, COREMINE Medical and other online dictionaries (e.g., Dictionary.com) were consulted to refine the search terms. The final search string was structured around four thematic components: digital education, dementia, caregiving and informal carers, along with their synonyms and variations.

We searched the following databases: PubMed, CINAHL, MEDLINE, ProQuest, Scopus, Embase, Cochrane, Web of Science, Wang‐Fang and CNKI. Additionally, Google Scholar was included to capture grey literature, while PADUA journal and Scopus citation tracking were employed to ensure coverage of the most relevant citations. Manual searches were performed on reference lists of identified articles to uncover additional studies not retrieved through database searches. The search strategy incorporated subject terms from thesaurus systems within the databases and free‐text words for flexibility. Broader and narrower terms from the databases' thesaurus were assessed for relevance and incorporated where applicable. Free‐text searches targeted keywords in article titles and abstracts to ensure comprehensive retrieval. Corresponding authors were contacted for full‐text access to articles that were unavailable online or through academic library systems. Table 2 displays the PCC (Population, Concept, Context) framework (Peters et al. 2015) used to guide the search and the detailed search strategy applied in PubMed.

**TABLE 2:** Search Strategy.

Category	Search terms
Population	‘Informal carer*’ OR ‘unpaid carer*’ OR ‘family carer*’ OR ‘spouse*’ OR ‘partner*’ OR ‘family member*’ OR ‘adult child*’ OR ‘friend*’ OR ‘neighbour*’
Context	‘Dementia’ OR ‘Alzheimer's disease’ OR ‘cognitive impairment’ OR ‘dementia care’ OR ‘caregiving’ OR ‘behavioural and psychological symptoms of dementia (BPSD)’
Concept	‘Digital education’ OR ‘digital training’ OR ‘e‐learning’ OR ‘online platform*’ OR ‘mobile app*’ OR ‘smartphone application*’ OR ‘telehealth’ OR ‘virtual reality’ OR ‘augmented reality’ OR ‘ICT’
PubMed search string	(‘Informal carer*’ OR ‘unpaid carer*’ OR ‘family carer*’ OR ‘spouse*’ OR ‘partner*’ OR ‘family member*’ OR ‘adult child*’ OR ‘friend*’ OR ‘neighbour*’) AND (‘Dementia’ OR ‘Alzheimer's disease’ OR ‘cognitive impairment’ OR ‘dementia care’ OR ‘caregiving’ OR ‘behavioural and psychological symptoms of dementia [BPSD]’) AND (‘Digital education’ OR ‘digital training’ OR ‘e‐learning’ OR ‘online platform*’ OR ‘mobile app*’ OR ‘smartphone application*’ OR ‘telehealth’ OR ‘virtual reality’ OR ‘augmented reality’ OR ‘ICT’)

### 2.4 Study Selection/Selection of Sources of Evidence

Identified references from the final database search were managed using Zotero (version 7.0) (Coar and Sewell 2010) to create a collaborative workspace for reviewers and streamline the screening process. Duplicates were removed systematically before initiating a three‐step screening process. First, one reviewer screened all titles to assess eligibility based on predefined inclusion and exclusion criteria, retaining articles with unclear eligibility for further evaluation. Second, the abstracts and keywords of the remaining articles were independently reviewed by two reviewers using the same criteria, with disagreements resolved through consensus. Articles meeting the eligibility criteria proceeded to the full‐text screening stage, where two reviewers independently assessed the retrieved full texts using Zotero's annotation and categorisation tools. Discrepancies were resolved through a consensus process guided by predefined inclusion criteria and, if necessary, reviewed by a third expert to ensure consistent application of criteria. The entire process, including reasons for exclusion, was meticulously tracked and documented using a shared spreadsheet. The PRISMA flow diagram (Figure 2) summarises the study selection process.

### 2.5 Data Extraction

A standardised data extraction table was developed to systematically summarise essential details from each study. This included the authors, publication year, country of origin, research objectives, study design, participant characteristics, intervention descriptions, primary outcomes and key findings (see Table 3). The feasibility of the digital education programmes was assessed by extracting data across five critical dimensions: Engagement, Ease of Use, Usability, Scalability and Satisfaction. This assessment was guided by the ISO 9241‐11:2018 usability framework (Interaction 2018), which defines usability as the extent to which a system achieves Effectiveness, Efficiency and Satisfaction in a specific context of use. In this study, the evaluation of usability incorporated these three core components and extended them to include Engagement, Ease of Use, Usability, Scalability and Satisfaction, thereby providing a comprehensive assessment of the digital education tools.

**TABLE 3:** Feasibility of Digital Dementia Education Tools.

Technology	Program	Feasibility				
		Engagement	Ease of use	Usability	Scalability	Satisfaction
Web‐based platform	STAR (Skills Training and Reskilling) E‐Learning Course (Hattink et al. 2015)		√	√	√	
	iSupport‐Portugal (Teles et al. 2022)	√	√	√	√	
	iSupport‐India (Baruah et al. 2021)	√	√	√		√
	Caregiver Skill Building Intervention (CSBI) (Farran et al. 2017)	√	√	√	√	
	RHAPSODY Online Support Program (Metcalfe et al. 2019)	√	√	√	√	
Video conferencing	Tele‐STAR (Lindauer et al. 2019)	√	√	√	√	
	Telehealth Behavioural Coaching Intervention (Steffen and Gant 2016)	√		√	√	
	Tele‐Savvy (Hepburn et al. 2022)	√	√		√	
	eHealth Psychoeducation Intervention (Singh Solorzano et al. 2023)	√		√	√	
	IHSS + ADRD Training Project (Yeh et al. 2023)	√	√	√	√	
	START Online (Kelly et al. 2024)	√	√	√	√	
APP	CARES (Caregiver Remote Education and Support) (Collins‐Pisano et al. 2024)	√		√		
	underst AID (Núñez‐Naveira et al. 2016)	√		√		
	Comprehensive Mobile Application Program (CMAP) (Park et al. 2020)	√	√	√		
	Estic amb tu—I'm With You (Romero‐Mas et al. 2021)	√	√	√		
	Mindfulness‐Based Self‐Compassion (MBSC) Support App (Goodridge et al. 2021)	√	√	√		
	STAV (Support for Family carers) (Dorell et al. 2022)	√	√			
SMS	CuidaTEXT (Perales‐Puchalt et al. 2024)	√	√	√		√

*Note:* Feasibility dimensions include: Engagement: retention rates and frequency of tool usage; Ease of use: navigation simplicity and user‐friendliness; Usability: practicality and functionality in caregiving scenarios; Scalability: capacity to reach broader audiences while maintaining effectiveness; Satisfaction: user‐reported relevance and perceived benefits.

Although related, feasibility and usability are distinct concepts. This review assesses feasibility using usability‐derived indicators reflecting user interaction, while implementation strategies refer to the broader processes supporting intervention delivery and integration. This distinction clarifies the different analytical dimensions examined.

The lead author conducted the initial data extraction, and a second author subsequently verified to ensure accuracy and reliability. Any disagreements during this process were resolved through consensus with a third author. Consistent with contemporary scoping review practices (Peters et al. 2021a), the formal quality appraisal was not conducted; however, all included studies were evaluated for methodological rigor and relevance based on predefined criteria, including clarity of research objectives, appropriateness of study design, transparency in data collection and analysis methods, and alignment of findings with the review objectives.

All authors agreed upon the final set of included studies, ensuring that each study demonstrated methodological soundness and was relevant to the research questions. A structured approach was adopted to present the results, beginning with an overview of study characteristics and then analysing how the included studies addressed the review objectives and research questions. Extracted data were presented in a clear and accessible format to facilitate understanding and interpretation.

### 2.6 Data Synthesis and Analysis

Following the recommendations of (Peters et al. 2015), the extracted data were synthesised using narrative and tabular formats to address the research questions systematically. Leveraging the authors' expertise in digital dementia education for informal carers, the analysis aligned educational objectives with the digital tool implementation methods.

Rodgers' six‐phase conceptual analysis method was chosen for its inductive nature, dynamic approach to evolving concepts, and suitability for clarifying and analysing related attributes (Rodgers et al. 2018). This approach aligns with the study's objective to identify and categorise key aspects of digital dementia education without relying on fixed definitions, ensuring adaptability to varying contexts and circumstances.

**FIGURE 1:**
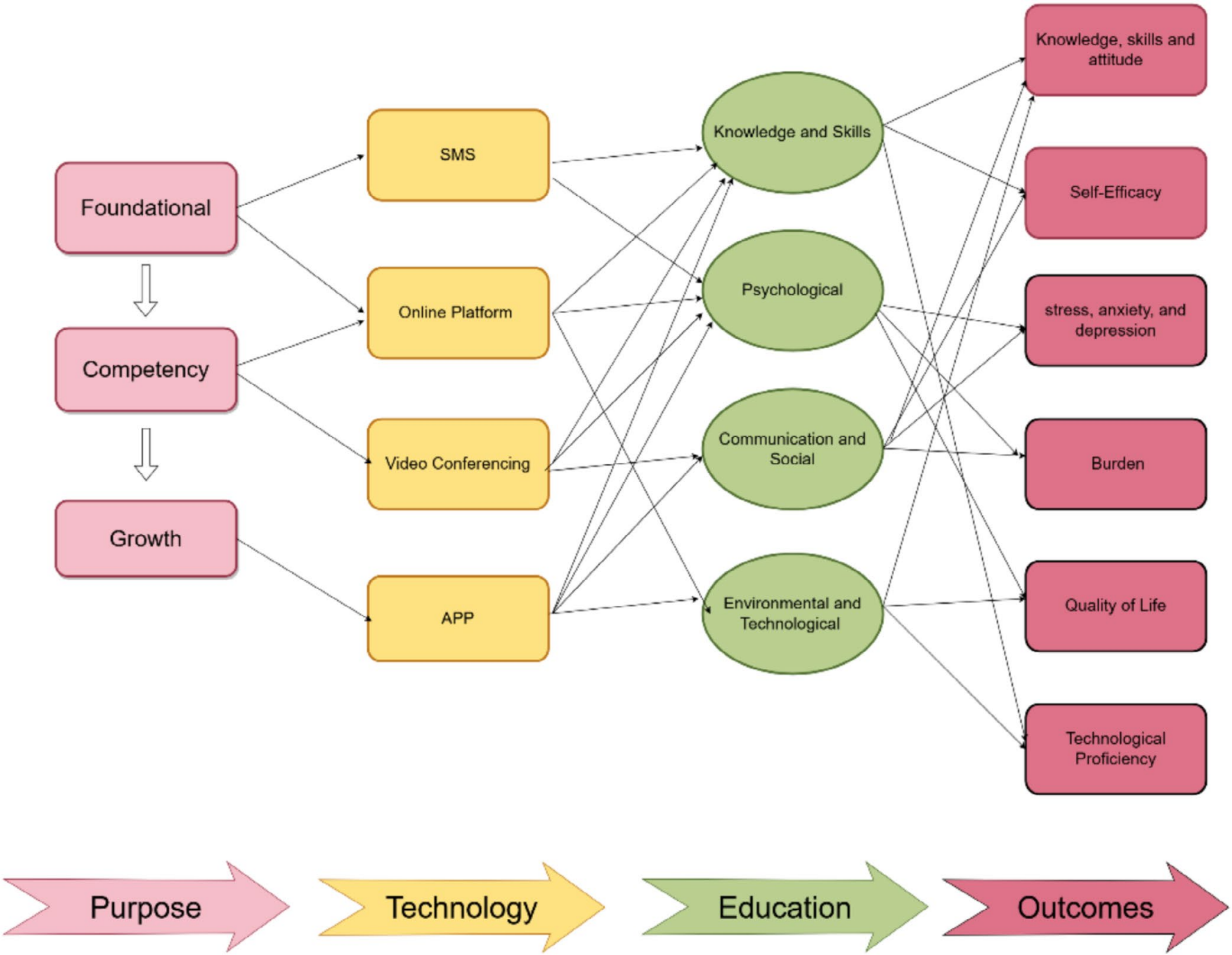
Conceptual framework for digital dementia education. [Colour figure can be viewed at wileyonlinelibrary.com]

The data were organised into four core dimensions: educational objectives, technological platforms, educational content and outcomes. These categories were synthesised into a theoretical framework for digital dementia education, as shown in Figure 1. This framework highlights the interconnections between educational goals, technological applications and outcomes, providing a structured guide for enhancing the capacity of informal carers in dementia care.

## 2 Results

### 2.1 Search and Selection Process

**FIGURE 2:**
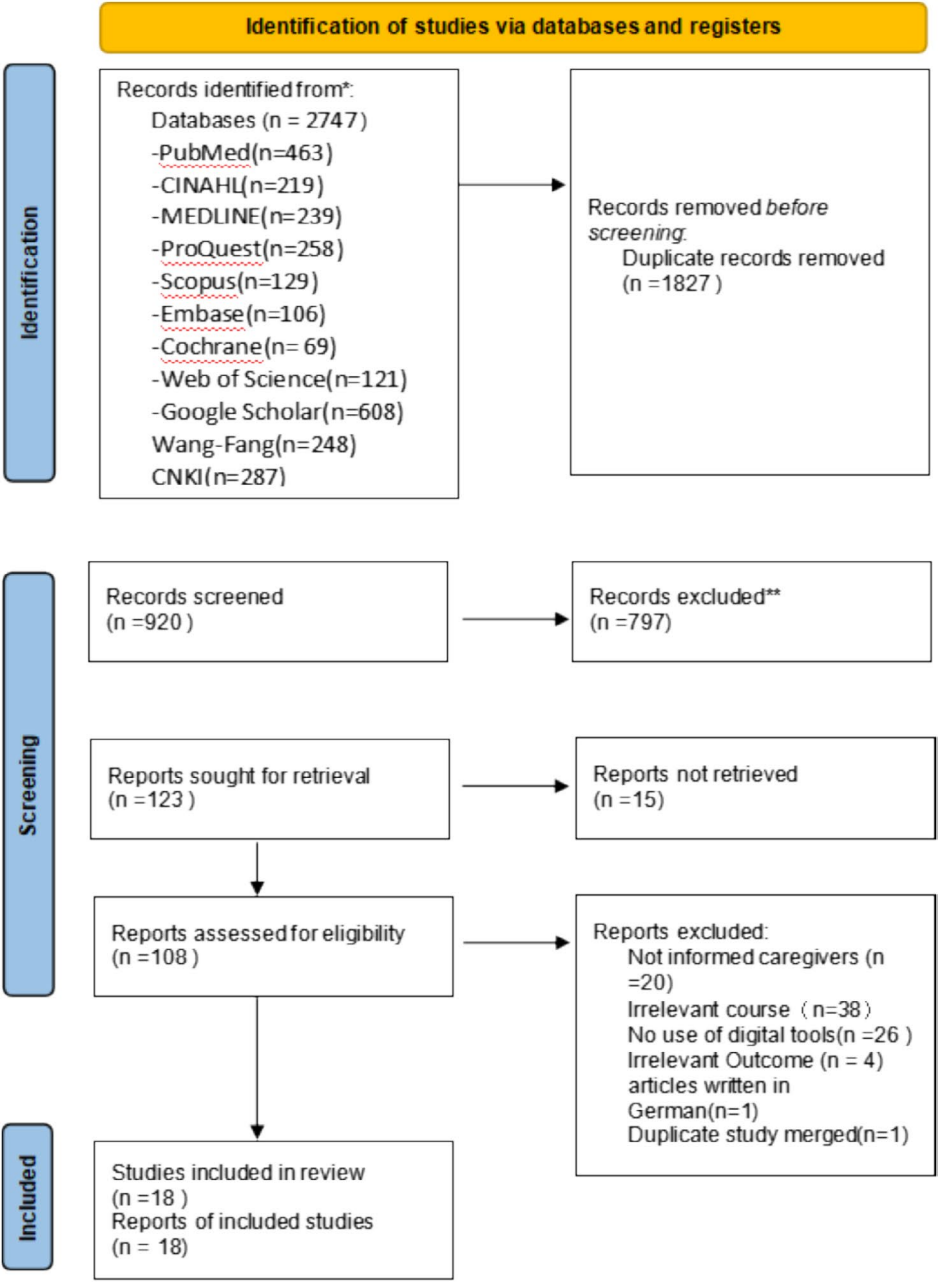
Search strategy and returns. [Colour figure can be viewed at wileyonlinelibrary.com]

A total of 2747 records were identified through systematic searches. After removing 1827 duplicates, 920 records remained for title and abstract screening, resulting in the exclusion of 797 articles. Of the 123 full‐text articles retrieved for eligibility assessment, 15 were unavailable. Among the 108 articles reviewed, 90 were excluded due to lack of focus on informal carers (*n* = 20), use of digital tools that did not include relevant dementia‐specific educational content (*n* = 38), absence of digital tools (*n* = 26), non‐aligned outcomes (*n* = 4) and German publication (*n* = 1). Two articles representing different phases of the same study were merged. Ultimately, 18 studies met the inclusion criteria and were included in the review. Although the search strategy included both English and Chinese publications, only English‐language articles met the inclusion criteria. The selection process is summarised in Figure 2.

### 2.2 Study Characteristics

The 18 studies included in this review were published between 2015 and 2024, with a notable concentration (66.67%) appearing in the past five years (2020–2024). These studies spanned 16 countries, with the United States accounting for the highest proportion (7 studies, 38.89%), reflecting the global emphasis on advancing digital interventions for informal dementia carers. Most studies employed quantitative methodologies, with six utilising randomised controlled trials (Collins‐Pisano et al. 2024; Hattink et al. 2015; Hepburn et al. 2022; Lindauer et al. 2019; Núñez‐Naveira et al. 2016; Park et al. 2020), six adopting pretest‐posttest designs (Baruah et al. 2021; Kelly et al. 2024; Singh Solorzano et al. 2023; Steffen and Gant 2016; Teles et al. 2022), and one conducting a longitudinal study (Goodridge et al. 2021). Qualitative insights were also captured through a descriptive study approach (Dorell et al. 2022). Informal carers, predominantly female family members or friends without formal caregiving training, constituted the primary participants, highlighting gendered caregiving dynamics. Detailed characteristics, including publication details, geographic focus, participant demographics, intervention types and key findings, are summarised in Tables S1 and S2.

### 2.3 Digital Tools for Dementia Education and Training

Online platforms (*n* = 5), such as STAR (Hattink et al. 2015; Metcalfe et al. 2019), are foundational tools that excel in delivering structured, self‐paced educational content focused on dementia symptoms, disease progression and caregiving principles. While their scalability and usability make them highly effective for knowledge dissemination, they lack real‐time interaction, limiting their capacity for emotional support. Video conferencing platforms (*n* = 6), including Tele‐STAR (Lindauer et al. 2019) and Tele‐Savvy (Hepburn et al. 2022), offer personalised, interactive sessions that enhance carers' problem‐solving abilities, self‐efficacy and stress management. However, the dependency on stable internet infrastructure limits accessibility in resource‐limited settings.

Mobile applications (*n* = 6), such as CARES (Metcalfe et al. 2019) and CMAP (Park et al. 2020), address both practical caregiving needs and emotional well‐being through multimedia resources, mindfulness exercises and peer‐support features. Despite their adaptability and interactive design, challenges related to interface complexity and continuous internet reliance impede sustained user engagement. In contrast, SMS services (*n* = 1), such as CuidaTEXT (Perales‐Puchalt et al. 2024), provide cost‐effective and accessible solutions, particularly for underserved populations with limited digital literacy. However, the absence of multimedia capabilities restricts their effectiveness in delivering comprehensive educational interventions.

Each platform demonstrates complementary strengths across key feasibility dimensions—engagement, ease of use, usability, scalability and satisfaction. Feasibility was assessed based on reported outcomes within the studies, including participant feedback, completion rates and user satisfaction metrics. Online platforms excel in scalability and foundational knowledge dissemination, video conferencing tools provide real‐time, personalised support and mobile applications offer flexibility while addressing emotional and practical needs. Though limited in interactivity, SMS services effectively reach populations with minimal resources. These findings highlight the importance of integrating diverse digital tools to create tailored, scalable and sustainable solutions that meet the multifaceted needs of informal carers at varying stages of their caregiving journey.

### 2.4 Educational Domains in Digital Dementia Interventions

Digital tools for dementia education targeting informal carers address four core educational domains: knowledge and skills (*n* = 17), psychological support (*n* = 13), communication and social interaction (*n* = 8), and environmental and technological adaptation (*n* = 7). These domains collectively reflect a comprehensive approach to meeting the multifaceted needs of carers while leveraging digital technologies for practical and emotional support. The alignment of digital tools with these educational domains highlights their complementary roles in addressing carers' evolving needs. While knowledge and skills form the foundation for caregiving proficiency, psychological support, communication and social interaction address emotional resilience and interpersonal dynamics. Environmental and technological adaptation ensures long‐term engagement and confidence in utilising digital tools, promoting a holistic, sustainable approach to carer education and support.

Knowledge and Skills constitute the foundational domain, focusing on equipping carers with essential information regarding dementia progression, symptom management and evidence‐based caregiving strategies. Tools such as STAR (Hattink et al. 2015), iSupport (Baruah et al. 2021; Teles et al. 2022) and CARES (Collins‐Pisano et al. 2024) effectively deliver structured, self‐paced content that enhances caregiving proficiency and knowledge retention. The accessibility and flexibility of these tools ensure widespread adoption; however, their lack of real‐time interaction can limit immediate emotional engagement for carers seeking direct support.

Psychological Support addresses carers' emotional and mental well‐being by targeting stress reduction, resilience building and self‐efficacy. Programmes like Tele‐Savvy (Hepburn et al. 2022), Tele‐STAR (Lindauer et al. 2019) and mobile applications such as CARES (Collins‐Pisano et al. 2024) and MBSC (Goodridge et al. 2021) integrate mindfulness exercises, emotional tracking and personalised coaching, significantly alleviating anxiety and emotional distress while enhancing coping mechanisms. Nevertheless, barriers such as digital literacy and technological infrastructure pose challenges, particularly in underserved settings (Goodridge et al. 2021).

Communication and Social Interaction focus on enhancing carer‐patient relationships and fostering peer support. Platforms such as CuidaTEXT (Perales‐Puchalt et al. 2024) and STAV (Dorell et al. 2022) facilitate real‐time messaging, group discussions and interactive sessions, enabling meaningful social engagement and emotional reinforcement. While these tools provide cultural adaptability and immediate support, maintaining sustained participation and fostering deep interpersonal connections remain areas of improvement. Environmental and Technological Adaptation emphasises empowering carers to optimise their caregiving environments and develop technological proficiency. Tools like understAID (Núñez‐Naveira et al. 2016) and Estic amb tu (Romero‐Mas et al. 2021) help carers confidently navigate digital platforms, ensuring resource accessibility, personalised learning and long‐term engagement.

### 2.5 Key Outcomes of Digital Dementia Interventions

This scoping review identifies six core outcomes of digital dementia education interventions for informal carers: knowledge, skills and attitude (*n* = 7), Self‐Efficacy (*n* = 6), stress, anxiety and depression (*n* = 15), burden (*n* = 13), quality of life (*n* = 10), and technological proficiency (*n* = 6). Improvements in knowledge, skills and attitudes are consistently observed across programmes due to structured, evidence‐based content delivered through self‐paced platforms such as CSBI (Farran et al. 2017) and RHAPSODY Online Support Program (Metcalfe et al. 2019). These tools effectively enhance carers' understanding of dementia progression, symptom management and practical caregiving strategies, fostering confidence and positive attitudes. Self‐efficacy gains result from personalised, interactive interventions like Tele‐STAR (Lindauer et al. 2019) and Tele‐Savvy (Hepburn et al. 2022), which provide real‐time guidance and tailored feedback, equipping carers to navigate challenging caregiving scenarios with greater competence and adaptability.

Reductions in stress, anxiety and depression reflect the significant focus on psychological support within digital interventions, particularly those integrating mindfulness exercises, emotional tracking and coping strategies, as seen in tools like CARES (Collins‐Pisano et al. 2024) and MBSC (Goodridge et al. 2021). These tools alleviate emotional distress, promote mental resilience and enhance coping mechanisms, particularly in high‐stress caregiving environments. Concurrently, burden reduction is facilitated by tools that simplify caregiving tasks, deliver timely support and combine educational content with emotional reinforcement, effectively alleviating physical and mental strain. Improvements in quality of life are closely linked to the holistic integration of practical caregiving strategies and mental health support, enhancing emotional well‐being, carer satisfaction and coping capacities. Finally, user‐friendly interfaces and guided navigation achieve technological proficiency outcomes, enabling carers to confidently engage with digital platforms and sustain long‐term adoption. Together, these findings demonstrate the multifaceted impact of digital interventions, highlighting their potential to deliver scalable, sustainable and comprehensive support that addresses informal carers' practical and emotional needs.

## 3 Discussion

This scoping review systematically examined the role of digital tools in dementia education for informal carers, synthesising evidence from 18 studies (Tricco et al. 2018).

### 3.1 Types of Digital Tools and Platforms

This review identifies four key types of digital tools—online platforms, video conferencing, mobile applications and SMS services that—cater to distinct aspects of dementia education for informal carers. Online platforms and SMS services effectively deliver foundational knowledge on dementia progression, symptom management and caregiving principles. Their scalability and ease of use make them particularly valuable in resource‐limited settings, where informal carers often lack formal training (Zimami and Darwish 2024). While these tools align with adult learning principles by offering structured, self‐paced education, their static design and lack of interactivity limit their ability to address caregiving's emotional and dynamic complexities. SMS services, despite their accessibility, are constrained by limited multimedia functionality, making them more suitable as complementary tools in hybrid models that combine foundational knowledge delivery with interactive features.

In contrast, video conferencing and mobile applications provide interactive, real‐time support that bridges the gap between theoretical knowledge and practical application (Kruger and Chowers 2020). Video conferencing enables experiential learning through simulated scenarios and real‐time feedback, enhancing informal carers' self‐efficacy and problem‐solving skills. Mobile applications extend this interactivity by integrating features such as mindfulness exercises, peer support and adaptive learning, addressing both the practical and emotional demands of caregiving (Chandran et al. 2022; Ryan et al. 2024). These tools adapt to carers' evolving needs and foster sustained engagement through personalisation and flexibility. Together, these digital tools create a layered approach to education, balancing accessibility with interactivity to support informal carers at different journey stages.

Despite the advantages of interactive digital tools, sustaining user engagement remains a critical challenge—especially for informal carers with limited digital literacy or those unfamiliar with technology‐based learning environments (Mueller et al. 2020). While mobile applications and video conferencing provide interactive elements, their effectiveness hinges on how well they accommodate users with varying levels of technical proficiency and confidence in using digital tools.

To enhance engagement, digital education platforms should prioritise intuitive, user‐friendly interfaces that minimise technical complexity. Simplified navigation, clear instructional design, and the use of universally recognisable icons can significantly reduce cognitive load, making digital tools more accessible to carers with limited technical experience (Chambers and Connor 2002). Additionally, hybrid models that integrate online education with offline support, such as telephone assistance or in‐person training, could further bridge digital literacy gaps.

Furthermore, ensuring cultural and linguistic adaptability in digital interventions fosters inclusivity and sustained engagement. Providing multilingual options, culturally relevant content and adaptable learning materials tailored to diverse caregiving contexts can help overcome barriers related to language proficiency and cultural perceptions of dementia care (LoBuono et al. 2021). Future research should investigate the effectiveness of these strategies in enhancing user retention and learning outcomes, ultimately optimising digital dementia education initiatives.

### 3.2 Educational Domains and Challenges

Digital interventions for dementia education systematically address the challenges faced by informal carers across four critical educational domains: knowledge and skills, psychological support, communication and social interaction, and environmental and technological adaptation. These domains provide a comprehensive framework to meet caregiving's practical and emotional needs.

The foundational domain, Knowledge and Skills, focuses on enhancing carers' understanding of dementia and equipping them with effective management strategies. Tools such as online platforms and SMS services deliver structured, evidence‐based content, enabling self‐paced learning and bridging initial knowledge gaps for new carers. However, their static nature limits adaptability as care demands evolve, highlighting the need for more interactive solutions to develop long‐term competency (Chambers et al. 2023; Enyoojo et al. 2024). Psychological Support addresses carers' stress and emotional burdens, critical factors contributing to burnout and reduced care quality. Video conferencing and mobile applications have demonstrated significant efficacy in integrating mindfulness exercises, emotional reinforcement and peer support, fostering resilience and coping mechanisms in alignment with Pearlin's Caregiver Stress Process model (Pearlin et al. 1990). These tools improve carer well‐being and indirectly enhance care outcomes for people with dementia. Communication and Social Interaction tools reduce isolation and strengthen carer‐patient relationships by facilitating peer connections and real‐time feedback. While these interventions align with social support theories (Birt et al. 2020; Parkinson et al. 2017), sustaining engagement remains challenging, particularly for carers with limited digital proficiency. Finally, Environmental and Technological Adaptation tools, though less commonly utilised, are essential in optimising caregiving environments and enhancing digital literacy, enabling carers to navigate and sustain digital interventions effectively over time.

### 3.3 Implementation Strategies and Outcomes

The success of digital dementia education programmes for informal carers hinges on three core strategies: personalisation, cultural sensitivity and technological adaptability. Personalisation aligns interventions with informal carers' varying needs and stages, from foundational knowledge acquisition to advanced skill‐building and emotional resilience (Turró‐Garriga et al. 2021). Tools like video conferencing and telehealth platforms offer tailored guidance and real‐time support, enhancing practical competency and self‐efficacy. Cultural sensitivity further improves engagement by adapting educational content to carers' socio‐cultural contexts and language preferences, as demonstrated in programmes like iSupport (Sani et al. 2024). Finally, technological adaptability ensures accessibility by incorporating user‐friendly designs and scalable platforms, enabling informal carers with diverse digital literacy levels to engage effectively with these interventions.

The outcomes of these strategies are profound, yielding measurable benefits across key caregiving dimensions. Digital tools enhance carers' knowledge, skills and attitudes, equipping them to manage dementia‐related challenges better. Interventions integrating Psychological support significantly reduce stress, anxiety and carer burden while improving emotional resilience, quality of life and overall well‐being (Feast et al. 2016; Palaza et al. 2024). Additionally, peer interaction tools foster social connectedness, mitigating isolation and reinforcing a sense of community among informal carers (Rapley et al. 2023). These results underscore the transformative potential of digital education programmes to provide scalable and sustainable solutions that address both practical and emotional aspects of caregiving, ultimately enhancing care quality and carer health.

### 3.4 Implications for Dementia Education and Training for Informal Carers

This review proposes a Conceptual Framework for Digital Dementia Education designed to address the dynamic and evolving challenges informal carers face as dementia progresses. By systematically integrating educational purposes, technological platforms, content domains and measurable outcomes, the framework provides a structured approach to developing, implementing and evaluating digital interventions (see Figure 1). Grounded in adult learning theories (Mukhalalati and Taylor 2019) and stress adaptation models (Pearlin et al. 1990), the framework was derived through thematic synthesis and is intended to conceptually organise implementation strategies without implying causal or sequential relationships.

The framework groups digital education tools into three interrelated functional domains—foundational, competency and growth—each reflecting distinct implementation strategies and support functions identified across the literature. Foundational tools, such as SMS services (Perales‐Puchalt et al. 2024) and basic online platforms (Hattink et al. 2015), provide essential knowledge about dementia symptoms, disease progression and caregiving principles. These tools are particularly effective for carers new to caregiving or in resource‐limited settings. Competency‐level tools, including video conferencing (Hepburn et al. 2022; Lindauer et al. 2019) and advanced online platforms (Baruah et al. 2021; Ryan et al. 2024), focus on enhancing carers' practical skills, problem‐solving abilities and communication strategies. Growth‐level tools, such as mobile applications (Dorell et al. 2022; Romero‐Mas et al. 2021), address higher‐order emotional and psychological needs, fostering resilience, reducing isolation and improving carers' quality of life (see Figure 1). These domains are not intended to reflect a sequential process, but rather a thematic synthesis of how implementation strategies can function across different caregiving contexts.

The proposed framework provides guidance for designing scalable, flexible and culturally responsive digital education interventions for informal carers. Rather than prescribing a linear developmental pathway, it highlights the potential to match diverse caregiving needs with suitable digital strategies, thereby ensuring that carers receive relevant support to strengthen their knowledge, caregiving skills and emotional coping capacity.

In addition to guiding the development of digital dementia education tools, the proposed framework also highlights opportunities for integrating these interventions into existing dementia care models, particularly through healthcare providers such as nurses. Foundational‐level tools, such as SMS services and online platforms, can be embedded into primary care and community‐based dementia screening programmes, ensuring that carers receive early‐stage educational support from healthcare professionals. At the competency level, video conferencing and advanced online platforms can be integrated into structured caregiver training programmes led by nurses, providing real‐time guidance, skill‐building opportunities and emotional support. Growth‐level tools, including mobile applications, can be incorporated into long‐term dementia care pathways, allowing nurses and healthcare teams to monitor carers' well‐being, facilitate peer support networks and promote resilience‐building strategies. By treating these domains as complementary rather than sequential, the framework promotes a flexible, needs‐based approach to intervention design that enhances accessibility, reinforces carers' competencies, and contributes to a more sustainable and integrated caregiving ecosystem.

### 3.5 Strengths and Limitations of the Study

This scoping review offers several notable strengths. First, the study systematically synthesises evidence and establishes a clear and structured theoretical framework for digital dementia education, providing a valuable reference for tailoring educational interventions to the evolving needs of informal carers. By incorporating multiple digital platforms, that is, online platforms, video conferencing, mobile applications and SMS services, this review demonstrates the versatility of digital tools in addressing key caregiving challenges, including improving knowledge, skills, psychological resilience and quality of life. Additionally, the study enhances the findings' generalisability by analysing a diverse range of interventions across various settings, showcasing the multifunctional capabilities of digital education tools. Using an established review framework ensured transparency, credibility and a systematic approach to evidence synthesis.

Nevertheless, this review also has several limitations. The heterogeneity of the included studies, such as variations in intervention design, duration and outcome measures, posed challenges for direct comparisons and limited the ability to draw overarching conclusions. Most studies were conducted in well‐resourced contexts, potentially limiting the applicability of findings to low‐ and middle‐income regions where access to technological infrastructure and digital literacy remains inadequate. For example, regional and cultural differences, as seen in studies on the iSupport program in Portugal and India, may result in varying educational outcomes, raising questions about the consistency of digital interventions across different cultural contexts. Another limitation is the lack of rigorous quality assessment for the included studies, which aligns with the scope of a scoping review but leaves the risk of bias and intervention validity unexamined. Additionally, many studies did not provide detailed descriptions of intervention components or their theoretical underpinnings, raising concerns about the replicability and integration of digital tools into broader carer education strategies. Lastly, including only English publications may narrow the scope of this review. Future research could focus on longitudinal evaluations, include underrepresented regions, and explore the scalability and inclusivity of advanced digital education technologies to address these gaps and further strengthen the evidence base for digital dementia education interventions.

## 4 Conclusion

This scoping review highlights the transformative potential of digital tools in dementia education for informal carers, presenting a structured theoretical framework that integrates educational purpose, technology, content and outcomes. By categorising digital interventions into the three functional domains of foundational, competency and growth, this review illustrates how a range of tools, including online platforms, video conferencing, mobile applications and SMS services can systematically address the evolving educational and emotional needs of informal carers. These tools deliver core knowledge on dementia care, enhance caregiving competencies through interactive and tailored support, and foster psychological resilience by mitigating stress, burden and social isolation. Reported outcomes such as improved knowledge, self‐efficacy, emotional well‐being and quality of life underscore the value of digital interventions in supporting carers as they navigate the complex demands of dementia care. However, challenges related to technological accessibility, cultural adaptability and sustained engagement remain evident, highlighting the need for ongoing refinement. Future research should focus on optimising intervention design, promoting equitable access, and assessing long‐term effectiveness to inform the development of sustainable, scalable and inclusive digital education solutions that improve outcomes for both carers and people living with dementia.

## Author Contributions

**Di Huang:** conceptualisation, methodology, data curation, formal analysis, writing – original draft, writing – review and editing, visualisation, validation, project administration. **Dongjun Wu:** conceptualisation, methodology, formal analysis, data curation, writing – review and editing. **Wendy Moyle:** conceptualisation, supervision, validation, writing – review and editing.

## Conflicts of Interest

The authors declare no conflicts of interest.

## Supporting information


Table S1.

Table S2.

## Data Availability

This scoping review did not generate or analyse any new datasets. All data utilised in this study are derived from previously published sources, which are fully cited in the reference list along with their respective DOIs. As no new data were created, deposition in a public data repository is not applicable.
