# Supplementary material for: Digital Dementia Education and Training for Informal Carers: A Scoping Review
Source: J Clin Nurs. 2025 May 28;34(10):3981–94. doi: 10.1111/jocn.17817 (PMC12409246; doi:10.1111/jocn.17817)
Supplement: Supplementary file 2 — Table S2. [file JOCN-34-3981-s001.docx]

Supplementary Table 2. Characteristics of included studies.

| **Reference** | **Aim** | **Design/Participants** | **Intervention** | **Outcome Variables** | **Key Findings** |
| --- | --- | --- | --- | --- | --- |
| (Hattink et al., 2015) Netherlands and UK | To evaluate the usability and effectiveness of the STAR (Skills Training and Reskilling) E-learning Course | RCT; 142 informal carers (Intervention: 79; Control: 69). Mean age in the Control group: 54.69 ± 14.36 years (Netherlands), 52.93 ± 11.43 years (UK) the mean age in the control group: 48.07 ± 9.11  (Netherlands) | Intervention Group: Access to the STAR portal for 2-4 months. The portal comprised 8 modules on dementia care (2 basic, 6 intermediate/advanced) and included peer and expert online communities for support and information exchange. Control Group: No specific dementia care training was provided during the study period. | Usability usefulness knowledge  Attitudes Empathy QoL（Quality of Life） Burden  Competence | - STAR was rated highly for usability and usefulness. - Improved person-centred care and attitudes toward dementia. - Increased empathy and perspective-taking. - Reduction in perceived competence in the intervention group. - Demonstrated potential as a user-friendly training tool. |
| (Lindauer et al., 2019) USA | To assess the efficacy of Tele-STAR in reducing burden and depression | Pretest-Posttest Design; 13 family carers Mean age: 67.1±7.4 years; caregiving duration: 3.4±2.5 years. Care recipients were mainly in the moderate stage of dementia (MoCA: 15.2 ±3.0) | 8 weekly videoconference sessions via a HIPAA-secure platform. Sessions guided carers through strategies for managing the emotional, cognitive, and physical impacts of distressing dementia-related behavioural symptoms using a manual and workbook. | Burden Depression QoL Behavioural symptoms Reactivity | - Tele-STAR reduced carer burden. - High satisfaction and adherence among participants. - Carers preferred the telehealth format over in-home training. - Feasibility and cost-effectiveness supported telehealth implementation. |
| (Collins-Pisano et al., 2024b) USA | To evaluate the usability, acceptability, and preliminary effectiveness of the CARES app | Mixed Methods Study (pre-post questionnaires and semi-structured interviews); 9 current informal carers (Mean age 67.3) and 3 trained informer carers | CARES App Intervention: Former family carers were trained as peer supporters to assist current carers via the CARES app. Over 2 weeks, interactions included messaging, video chat, and access to educational resources within the app. | Usability Acceptability Burden Stress Strain levels | - High usability ratings by current carers. - Feasibility of using trained former carers for peer support. - Identified technical issues (messaging delays and notification problems). - Suggested improvements include telephone features and enhanced notifications. |
| (Núñez-Naveira et al., 2016) Denmark, Poland, Spain | To evaluate the technical, pedagogical, and psychological impact of the understAID ICT platform | RCT;61 carers (Intervention: 30; Control: 31; age 25–88) | A 3-month trial using the understAID application included education, skills training, social networking, and daily reminders. | Usability,  satisfaction,  depressive symptoms, competence,  caregiving satisfaction | - Reduced depressive symptoms among users. - Significant benefits for inexperienced carers. - Low satisfaction scores highlight the need for technical and pedagogical improvements. |
| (Park et al., 2020) South Korea | To evaluate the impact of a comprehensive mobile application program (CMAP) on carer fatigue, sleep, burden, and BPSD | Nonequivalent control group pretest-posttest design;26 carers (≥6 hours/daycare for 1+ month; Mean age: 57.2 ± 8.5 years) Care recipients were predominantly in the moderate-to-severe stages of dementia. | A 4-week CMAP intervention, including education on dementia, interventions, communication skills, coping methods, and bulletin boards. | Fatigue Sleep  Burden BPSD  Stress | - Significant improvements in carer fatigue and burden. - No significant impact on stress or sleep. - Demonstrated potential for managing BPSD in caregiving contexts. |
| (Hepburn et al., 2022) USA | To evaluate Tele-Savvy for improving psychological well-being and caregiving mastery | Three-armed, waitlist, randomised trial design; 261 carers (Intervention: 96; Control: 111; Usual care: 54) Mean age: 64.6 years (range: 29–89 years) | Active intervention group: Tele-Savvy program over 43 days with 7 synchronous sessions and 36 asynchronous video lessons. Attention control group: Similar format program focusing on healthy living. Usual care group: No intervention provided. | Psychological well-being  Mastery Burden Anxiety | - Improved emotional well-being and caregiving mastery. - Burden and anxiety levels remained unchanged. - Scheduling and internet access were noted as key barriers. |
| (Singh Solorzano et al., 2023) Italy | To assess Health psychoeducation’s impact on the burden and neuroendocrine markers | Single-arm pre-post study design; 41 carers (Mean age 57.15 years; 78% female) | Based on the 'Savvy Carer Program' and the 'Medway Carers Courses' models, the intervention consisted of 8 weeks of online synchronous group sessions, 2 hours per week, with invited experts introducing specific topics, led by a psychotherapist experienced in dementia care. | Self-efficacy Anxiety Depression Burden | - Increased self-efficacy in managing dementia-related challenges. - Reduced anxiety and burden post-intervention. - No significant changes in depressive symptoms or cortisol levels. - High participant satisfaction. |
| (Romero-Mas et al., 2021) Spain | To evaluate the impact of participation in a Virtual Community of Practice (VCoP) on the quality of life of family carers of individuals with Alzheimer's disease. | Pretest-posttest quasi-experimental design;38 carers (Mean age 56; 79% female) | 10-month VCoP intervention via the app "Estic amb tu – I’m With You" (online discussions and face-to-face expert-moderated sessions) | QoL Functional deterioration,  eHealth literacy | - Improved anxiety and QoL, particularly in the environmental domain. - Protective effect against QoL decline despite care recipient deterioration. - Positive app experiences among participants. |
| (Teles et al., 2022) Portugal | To assess the feasibility of online training and support programmes for dementia carers and to explore how the intervention and control groups compare over time on well-being outcomes. | In the mixed methods study (randomised controlled trial and semi-structured interviews); 42 carers participated (Intervention: 21; Control: 21).  Mean age: 53.6 ± 13 years | Intervention Group: accessed the iSupport-Portugal program, a self-guided online platform with 5 modules and 23 lessons on topics such as understanding dementia, communication, and self-care. Control Group: received an education-only e-book covering dementia and caregiving information. The intervention period lasted 6 months. | carer burden Depression  Anxiety Self-efficacy  QoL | - Improved anxiety and QoL among the intervention group. - Higher dropout rates among younger carers and those with elevated anxiety. - Recommendations for multimedia and personalisation. |
| (Baruah et al., 2021) India | To assess the feasibility and preliminary effectiveness of an online training and support program for carers of people with dementia in India. | RCT; 151 carers (Intervention: 74; Control: 77). Mean carer age: 46.5 ± 14.1 years | Intervention Group: received access to the iSupport program, an online, interactive platform with 23 lessons across five themes: understanding dementia, caregiving, self-care, everyday care, and managing behavioural changes.  Control Group: received an education-only e-book on dementia care. The intervention lasted 3 months. | Subjective burden Depression Attitude  Self-efficacy | - Improved person-centred attitudes toward persons with dementia. - Low engagement and retention rates. - No significant impact on depression or burden. |
| (Yeh et al., 2023) USA | To enhance dementia knowledge and self-efficacy of In-Home Supportive Services (IHSS) carers through online training. | longitudinal design; 92 carers (Mean age: 69 years; 52% married; 93% female) | 35-hour online training program delivered via Zoom; covered dementia understanding, caregiving strategies, and BPSD. | Knowledge Self-efficacy Distress Depression  Satisfaction | - Significant improvement in dementia knowledge and self-efficacy. - High satisfaction post-training (94%). - Skills and knowledge retained at 3-month follow-up. - Demonstrated scalability to other regions. |
| (J Farran et al., 2017) USA | To develop and test a web-based carer Skill Building Intervention (CSBI) | pretest-posttest design;100 family carers (49% spouses, 46% adult children/in-laws) Mean age: 59.2 ± 12.4 years | The intervention lasted 12 weeks. Carers completed six interactive modules designed to provide immediate feedback on caregiving scenarios. The modules were accessible anytime with internet access. | carer Skills Burden Depressive symptoms,  Positive affect | - Improved caregiving skills at 6 and 12 weeks. - Reduced depressive symptoms and increased positive affect at 6 weeks. - High satisfaction with content and module relevance. |
| (Goodridge et al., 2021) Canada | To assess the feasibility and preliminary efficacy of a mobile app-based mindfulness-based self-compassion (MBSC) program for supporting carers of people with dementia. | Mixed methods study (pre-post questionnaires and semi-structured interviews); 57 carers (Mean age: 76.3 years; spouses/children/relatives) | 12-week mindfulness-based self-compassion app (podcasts, meditations, daily emotional assessments) | carer burden Coping styles Emotional well-being | - User-friendly app with high convenience. - Improved emotional well-being post-intervention. - Shift from emotion-focused to adaptive coping strategies. - Demonstrated feasibility and demand for web-based MBSC tools. |
| (Kelly et al., 2024) Australia | To assess the adaptation and feasibility of the START online multicomponent intervention for Australian carers of people with dementia. | Two-armed, randomised controlled trial; 28 carers (18 START; 10 CBT). Mean age: 63.5 ± 10.4 years. Care recipients were in various dementia stages. | START Program Group (N=18): Eight weekly telehealth sessions on dementia education and coping skills  CBT Group (N=10): Standard Cognitive Behaviour Therapy in a University Clinic | Feasibility Anxiety Depression Burden | - High feasibility and satisfaction with the START program. - Good engagement and completion rates. - Improved mood among some carers. - Limited reduction in burden, especially for those with high baseline burden. - Telehealth satisfaction is comparable to face-to-face sessions. |
| (Perales-Puchalt et al., 2024) USA | To evaluate bilingual CuidaTEXT intervention for Latina carers | One-arm pre-post-intervention trial design; 24 carers (Mean age: 52.6 years; 83% women) | Six-month bilingual text-message program tailored to needs (education, problem-solving, resources, live chat) | Feasibility Distress  Depression  Competence  Knowledge Coping | - High follow-up completion (83.3%) and thorough engagement with messages (85.7%). - High satisfaction with intervention. - Reduced distress and depression. - Improved dementia knowledge, competence, and coping strategies. |
| (Steffen and Gant, 2016) USA | To assess the differential impact of two telehealth programmes for women caring for an older adult with a neurocognitive disorder. | Single-blind, randomised controlled trial; 74 women carers (spouses/adult/children) cohabitating with relatives diagnosed with neurocognitive disorders.  Mean age of carers: 60.3 ± 10.8 years | Behavioural Coaching Condition: 14-week program with video instruction, workbooks, and weekly telephone coaching on behavioural management, relaxation, and scheduling pleasant events. Basic Education and Support Condition: 14-week program with a written care guide and biweekly telephone support on dementia care information and guidance. | Depression Self-efficacy | - The Behavioural Coaching Condition significantly reduced depressive symptoms and negative mood states. - Improved self-efficacy in managing patient behaviours. - At the **6-month follow-up**, the Behavioural Coaching group maintained improvements, but no statistically significant difference was observed between the two groups. |
| (Metcalfe et al., 2019) England, France, Germany | To assess the acceptability of online support programs for young-onset dementia | Mixed methods study (pilot unblinded randomised waitlist controlled trial and semi-structured interviews); 61 carers (Intervention: 30; Control: 31).  Mean carer age: 57.4 ± 10.2 years | Intervention group: received immediate access to a 6-week online programme. Control group: delayed access.   seven modules on young-onset dementia | Acceptability  Well-being Stress  Burden Competence | - Acceptable and useful for 70% of participants. - Reduced stress and negative reactions to memory symptoms. - Cost-effective program suitable for broad dissemination. - 85% of participants intended to use the program in the future. |
| (Dorell et al., 2022) Sweden | To explore the experiences of family carers receiving professional support through a mobile app and its usage. | Qualitative descriptive study: 15 family carers (7 women, 5 men). Participants were 18 years or older, accompanied a family member with dementia to an outpatient cognitive clinic, and had access to a mobile device with internet | 8-week STAV app program (chat, mindfulness exercises, personal diary, weblinks) | Experiences of using the mobile app, its different features, and perceived usefulness and satisfaction. | - Easy access to professional support via mobile app. - Chat feature valued for direct communication. - Mixed views on group chat; some preferred private communication. - Mindfulness exercises are underutilised due to caregiving demands. - Technical barriers highlighted the need for user support. |
